# Multivariate analysis of microarray data: differential expression and differential connection

**DOI:** 10.1186/1471-2105-12-42

**Published:** 2011-02-01

**Authors:** Harri T Kiiveri

**Affiliations:** 1 CSIRO Mathematics Informatics and Statistics, The Leeuwin Centre, 65 Brockway Road, Floreat, Western Australia

## Abstract

**Background:**

Typical analysis of microarray data ignores the correlation between gene expression values. In this paper we present a model for microarray data which specifically allows for correlation between genes. As a result we combine gene network ideas with linear models and differential expression.

**Results:**

We use sparse inverse covariance matrices and their associated graphical representation to capture the notion of gene networks. An important issue in using these models is the identification of the pattern of zeroes in the inverse covariance matrix. The limitations of existing methods for doing this are discussed and we provide a workable solution for determining the zero pattern. We then consider a method for estimating the parameters in the inverse covariance matrix which is suitable for very high dimensional matrices. We also show how to construct multivariate tests of hypotheses. These overall multivariate tests can be broken down into two components, the first one being similar to tests for differential expression and the second involving the connections between genes.

**Conclusion:**

The methods in this paper enable the extraction of a wealth of information concerning the relationships between genes which can be conveniently represented in graphical form. Differentially expressed genes can be placed in the context of the gene network and places in the gene network where unusual or interesting patterns have emerged can be identified, leading to the formulation of hypotheses for future experimentation.

## Background

Differential expression analyses of microarray data [1] typically ignore any correlation between genes. In this paper we consider a model for microarray data which explicitly includes correlation structure between genes and we explore its implications for estimation and significance testing.

The model presented below involves the use of large sparse inverse covariance matrices [2,3] and an associated graphical representation of the inverse covariance matrix [4] which we use to encode the (linear) relationships between genes. We discuss the estimation of mean and covariance structure, including the problems of determining the pattern of zeroes in the inverse covariance matrix and fitting the matrix to data once the pattern has been determined. For the purposes of hypothesis testing we will describe a permutation procedure [5] to test the significance of a hypothesis overall as well as a breakdown into components involving differential expression and "differential connection".

## Results

### The model

Consider p expression measurements, measured on n individuals, arranged in an n × p data matrix *X*. In addition, each individual is subject to known "treatments". We assume that individual i is subject to a treatment (combination) given by row i of an n × r design matrix *D *and that the p gene expression measurements for each individual have common covariance matrix Σ. If we denote the operation of making a vector from a matrix row by row by vec{...}, then we can write the joint model for this data set as

(1) vec{X} ∼N(vec{DB},I⊗Σ)

where *B *is an r × p matrix of treatment effects and ⊗ denotes the tensor product. From (1) it is easily seen that the i^th ^row *X_i _*and j^th ^column *X_j _*of *X *have distributions

(2) $$\begin{array}{l} X_{i.}\;∼\;N\left(D_{i.}B,\Sigma \right)\\ X_{.j}\;∼\;N\left(DB_{.j},\;\sigma_{Jj}I\right) \end{array}$$

Where *σ_jj _*is the j^th ^diagonal element of Σ, *D*_*i. *_is the i^th ^row of *D *and *B_.j _*is the j^th ^column *B*. For those more familiar with stacking matrices column by column, see the Methods section. From (2) we see that each row of X has a multivariate normal distribution with mean structure dependent on the treatment and covariance structure defined by Σ. Similarly each column of X has a mean structure defined by the design matrix *D *and variance structure a multiple of the identity matrix, a typical structure in regression models.

In the above we consider Σ to be a function of some parameters *θ*. In particular, we will assume that Σ^-1, ^the inverse covariance matrix, is sparse and the parameters *θ *correspond to the nonzero elements of the inverse covariance matrix. Such matrices define (sparse) Gaussian graphical models (sometimes referred to as covariance selection models), see for example [3] and [4]. These models have conditional independence interpretations. Writing X_i _for the i^th ^variable (gene), X_-i _for the vector of the remaining variables, μ_i _for the mean of variable i and σ^ij ^for the i,j^th ^element of the inverse covariance matrix Σ^-1 ^we have

(3) $$\begin{array}{l} E\left\{X_{i}|X_{-i}\right\}=\mu_{i}-\sum_{j\neq i}\left(\frac{\sigma^{ij}}{\sigma^{ii}}\right)\left(X_{j}-\mu_{j}\right)\\ V\left\{X_{i}|X_{-i}\right\}={\left(\sigma^{ii}\right)}^{-1} \end{array}$$

Where *E *denotes conditional expectation and *V *conditional variance.

The interpretation of zero elements of the inverse being equivalent to regression coefficients (*β_ij _*= -(*σ^ij^*/*σ^ii^*)) being zero makes this class of models attractive for analysing microarray data as it provides useful information about the (linear) interrelationships between genes. We define the set of neighbours of variable (gene) i to be the set of variables with non zero regression coefficients in (3) above.

**Figure 1:**
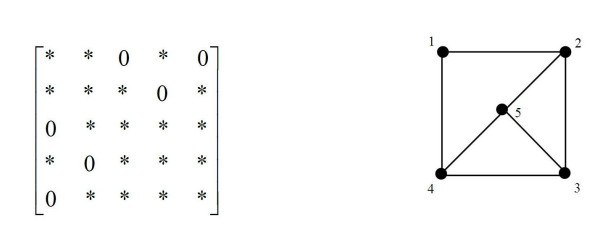
**Example zero pattern in inverse covariance matrix and corresponding graphical representation**. (* denotes non-zero).

An undirected graph can be associated with any pattern of zeros in the inverse covariance matrix by the relation: there is an edge between vertices i and j if and only if *σ^ij ^*≠ 0, where vertex i denotes variable *X_i_*. An example of this is given in Figure 1 below.

The cliques, maximal sets of vertices which are all connected, of the graph are {1, 2}, {1, 4}, {2, 3, 5}, and {3, 4, 5}. From this graph we can see for example that the regression of variable 1 on the rest has nonzero regression coefficients for variables 2 and 4.

Unlike traditional microarray analysis [1], the above model will enable the analysis of microarray data in a way which makes use of correlations between genes and respects the idea of genes being connected in a network.

Note that the model in this section is an example of a (very high dimensional) mean linear hierarchical mixed graphical model as defined in [6,7]. See also the supplementary information [Additional file 1].

### Parameter estimation

To implement the model described in section 2 above we require estimates for the matrix parameter *B *and for the non-zero entries of the inverse covariance matrix Σ^-1^. We discuss these topics below.

### Estimating parameters in the mean structure

We use maximum likelihood to estimate the parameter matrix *B*. From (1), the log likelihood function is

(4) $$\begin{array}{l} L=f\left(\Sigma^{-1}\right)-0.5^{*}vec{\left\{X-DB\right\}}^{T}\left(I⊗\Sigma^{-1}\right)vec\left\{X-DB\right\}\\ \;\;\;=f\left(\Sigma^{-1}\right)-0.5^{*}trace\left\{\left(X-DB\right)\Sigma^{-1}{\left(X-DB\right)}^{T}\right\} \end{array}$$

where f denotes a function of Σ^-1 ^independent of *B*. Using matrix calculus [8,9], and differentiating this expression with respect to *B *we obtain

(5) $$\partial L/\partial B=-vec\left\{D^{T}\left(X-DB\right)\Sigma^{-1}\right\}$$

It follows that the maximum likelihood estimate of *B *is

(6) $$\hat{B}={\left(D^{T}D\right)}^{-1}D^{T}X$$

From equation (6) we see that the estimates of the columns of *B *are simply obtained by individual regression of each column of *X *on the design matrix *D*.

### Estimating parameters in the variance structure

The discussion in this section involves computationally intensive methods aimed at discovering (linear) relationships *between *genes. It is precisely this information which is ignored in traditional microarray analysis.

In principle, estimating the parameters in Σ^-1 ^involves two computationally demanding problems. Firstly, identifying the pattern of zeroes, and secondly, estimating the inverse covariance matrix for a given pattern of zeroes. The difficulties are caused by the high dimensionality of the data e.g. the number of genes can be of the order of p = 20000 or more. For example, with p = 20000 there are approximately 200 million unique elements in Σ^-1 ^to work with. Without care, attempting to do these tasks can result in very large memory requirements and very long cpu times. In this high dimensional setting standard methods for these tasks become very slow or even infeasible. However, some progress can be made and we begin by discussing methods for determining the pattern of zeroes in the next section below.

### Determining the pattern of zeroes

There are two common methods for determining the pattern of zeroes for a given a data set. The first method involves computing p individual regressions of each variable on the remaining variables. This is intuitively reasonable given our earlier discussion about the interpretation of elements of the inverse covariance matrix in terms of regression coefficients, see Equation (3). The regression method used for the individual regressions could incorporate a sparsity penalty, as in the zero pattern finding method in [10], or simply be some form of consistent stepwise variable selection, either using a forward stepwise variable selection method as in [11], or a combination of forward and backward selection with a modified BIC criterion [12,13]. A simple forward stepwise regression algorithm is described in the supplementary information [Additional file 1]. Major advantages of these methods in the high dimensional setting are the ability to use existing software and to easily distribute the problem over multiple processors. However some care is required to avoid overfitting.

A second class of methods is maximum likelihood estimation with L1 (more generally sparsity) constraints on the elements of sigma inverse. These methods accomplish simultaneous model selection and fitting, see for example [14,15] and [16]. A likelihood *approximation *with L1 constraint is considered by [17] and [18]. Note that if we use these methods as a pattern selector, we still may wish to compute maximum likelihood estimates of parameters for the selected pattern of zeroes.

Of the above methods, the method of [15] is a good benchmark for problems with a few thousand variables. However, this second class of methods is not well suited for data with tens of thousand of variables or more, both from the viewpoint of memory requirements and cpu time. The method of [15] transforms the problem into a series of L1 regressions which are solved efficiently via a coordinate ascent procedure. Unfortunately, experiments have shown that, in the case of very large numbers of variables, the overhead in creating these Ll regression problems is too large and the cyclic updating procedure can converge very slowly for problems with realistic structure, see for example (Kiiveri H and deHoog F: Fitting very large sparse Gaussian graphical models, submitted). The methods of [15], [17] and [18] are implemented in R (R Development Core Team (2009)) as packages glasso, space and spice. They clearly are not designed for very high dimensional problems as they use dense matrix computations. In addition, convergence for a regularisation parameter value of 0 can be a problem, in particular when the sample covariance matrix is not full rank. As a consequence, high dimensional problems will not run on a desktop computer, and there are other problems as well. For example, the current implementation of the likelihood approximation method *space *doesn't allow specification of a pattern of zeroes in Σ^-1 ^a priori. Individual iterations for a fixed regularisation parameter must be done instead and can be *very *slow for models with large numbers of variables. The specification of a sparse model can also be clumsy,requiring vectors identifying *all *zero elements.

However, in the interest of computing speed, simplicity and easy accessibility, we propose using an efficient forward stepwise algorithm as implemented in the R package lars [19] coupled with a modified BIC criteria. One modified BIC criterion [13] is

(7) $$BIC_{r}=n\log\left({\hat{\sigma }}^{2}\left(k\right)\right)+k\log\left(n\right)+2\gamma (\log\underset{k}{\left(p\right)})$$

where s *σ*^2 ^(*k*) is the maximum likelihood residual variance estimate for a linear regression model with k predictors, 0 ≤ *γ *≤ 1 and $\left(\begin{array}{l} p\\ k \end{array}\right)\;=\;p!/\left(\left(p-k\right)!k!\right)$ denotes the number of subsets of size k when there are p variables to chose from. Note that when *γ*= 0, (7) corresponds to the usual BIC. In the case of very many more variables than observations the recommended value of *γ *is one. We also compared an alternative version of BIC defined by

(8) $$BIC_{2p}=n\log\left({\hat{\sigma }}^{2}\left(k\right)\right)+k2\log\left(p\right)$$

see [12].

To determine the zero pattern in Σ^-1 ^we adopt a simple strategy. For each gene we do forward variable selection up to a pre-specified model size kmax (see the discussion), considering all other genes as potential predictors i.e. for each column i in turn, we use forward selection to chose predictors for the i^th ^column of the expression matrix X from amongst the remaining columns using a forward stepwise algorithm. We then choose the model size with minimum modified BIC as in (7) or (8). Each of these regressions contribute to a sparse neighbour matrix *A *defined by

$${\text{A}}_{ij}\text{=}\{\begin{array}{ll} 1, & \text{if}\;\text{gene}\;\text{j}\;\text{chosen}\;\text{as}\;\text{a}\;\text{predictor}\;\text{of}\;\text{gene}\;\text{i}\\ 0, & \text{otherwise} \end{array}$$

Finally the zero pattern is determined by computing *N = A + A^T ^*and setting all diagonals and nonzero entries to 1 in the resulting matrix.

Clearly we can use other regression strategies such as L1 constrained regression in a similar manner, however they will typically be at least 2 to 3 times slower in terms of computing time if cross validation is used to select the regularisation parameter.

### Simulation study

We conducted a simulation study to explore the properties of the forward stepwise procedure outlined above. Choices are somewhat constrained by the difficult tasks of simultaneously controlling the median neighbourhood size, signal to noise ratios and positive definiteness for very large matrices. The simulations are also cpu intensive.

Sparse inverse covariance matrices with known structure were generated as follows.

1. Generate the p × p neighbour matrix A by randomly selecting q elements of each row to be non-zero. Compute the matrix *N = A + A^T^*, set the diagonals and all non-zero entries of the resulting matrix to one. Note that as a result of the last step, neighbour sizes can and do vary from the selected q. The parameter q can be varied to control the median neighbour size. i.e. the median number of non-zero entries in a row of Σ^-1 ^excluding the diagonal.

2. Construct Σ^-1 ^as follows. Set the non-zero upper triangular elements of Σ^-1 ^to be the same as those of *N*. Generate each individual non-zero value from a uniform distribution over the interval [-1,0.5]∪[0.5,1]. (Note that these intervals were chosen in order to exclude "small" values in Σ^-1 ^Multiplying the resulting matrix by a scalar will increase the range of the parameters but doesn't really add interesting structure). Finally, symmetrise the matrix.

3. Set the i^th ^diagonal of Σ^-1 ^to be the sum of the absolute values of the elements in the i^th ^row excluding the diagonal, times a constant d, 0 < d ≤ 1, chosen so that the resulting matrix is still positive definite. This was done by stepping down from 1 in steps of .05 until the matrix was no longer positive definite. The smallest value for which the matrix was positive definite was chosen. The motivation for this step is to improve the typically poor signal to noise ratios in diagonally dominant matrices.

The simulation considered all combinations of the three factors

• Median neighbour size m = 5, 10

• Number of genes p = 5000, 10000 and 20000

• Sample size n = 100, 200 and 500

For each combination of median neighbour size m and number of genes p, inverse covariance matrices were constructed as described above. Multivariate normal samples of sizes of 100, 200 and 500 were then drawn from each of these. Sparse matrix calculations are essential for this step. The zero pattern finding procedure was applied to each data set with different versions of BIC and the resulting structure compared to the known structure. This entire process was repeated 10 times.

A typical covariance matrix for m = 5 had 90% of neighbour sizes in the range 3 to 8 with maximum size 15, and for m = 10, 90% of the neighbour sizes were in the range 8 to 15 with maximum neighbour size 21.

**Figure 2:**
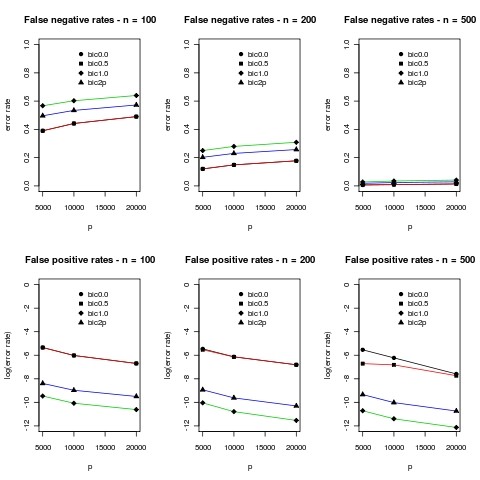
**Results of simulations for m = 5**. Mean error rates over 10 simulations are plotted. The standard deviations were typically less than 5% and difficult to distinguish on the plots so they were omitted. In the plots bic0.0 corresponds to *γ *= 0 in (7), bic0.5 to *γ *= 0.5, bic1.0 to *γ *= 1.0 and bic2p corresponds to BIC defined by equation (8). We use a log scale for the false positive rates because the number of zero entries is so large that it is difficult to make sense of the numbers in the original scale.

Results for the case m = 5 are given in Figure 2 below.

We note a dramatic improvement in the false negative rate as sample size increases. The results for the usual BIC and BIC_0.5 _are very similar and consistently produce the best false negative rates. They also produce similar false positive rates, however they are now consistently worse compared to BIC_1.0 _and BIC_2p_.

**Figure 3:**
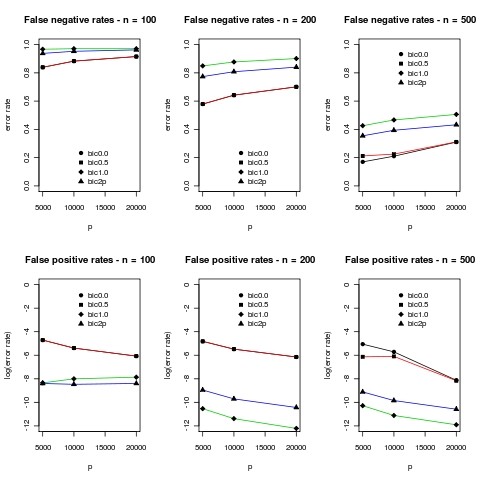
**Results of simulations for m = 10**. Mean error rates over 10 simulations are plotted. The standard deviations were typically less than 5% and difficult to distinguish on the plots so they were omitted. In the plots bic0.0 corresponds to *γ *= 0 in (7), bic0.5 to *γ *= 0.5, bic1.0 to *γ *= 1.0 and bic2p corresponds to BIC defined by equation (8). We use a log scale for the false positive rates because the number of zero entries is so large that it is difficult to make sense of the numbers in the original scale.

Results for m = 10 are given in Figure 3 below

The same qualitative patterns as in Figure 2 are evident in Figure 3. Note however that the false negative rates are much higher here for m = 10. This can be partly explained by differences in the number of observations per parameter ≈ n/m in each regression relationship, this ratio being twice the size when m = 5. The remaining difference may be explained by differences in the signal to noise ratios for the two cases, a factor which appears to be difficult to control precisely when generating these large covariance matrices. For an interesting discussion of the effect of the relationship between m and n and p and n in the ability to recover regression relationships in high dimensional data see [20].

Tables 1 and 2 below give the mean confusion matrices and standard deviations in the case m = 10, p = 20000 and n = 500 for BIC0.0 and BIC1.0. Results for other combinations are qualitatively similar.

**Table 1:** Mean confusion matrix for m = 10, p = 20000 and n = 500 using BIC_0.0_

true	no edge	Predicted edge
no edge	199830772.5	59249.8
	(227.8)	(229.7)
edge	31001.4	68976.3
	(117.3)	(119.7)

Standard deviations in brackets.

**Table 2:** Mean confusion matrix for m = 10, p = 20000 and n = 500 using BIC_1.0_.

true	no edge	Predicted edge
no edge	199888674.5	1347.8
	(39.9)	(38.8)
Edge	50587.9	49389.8
	(156.7)	(158.4)

Standard deviations in brackets.

It is clear from these tables that the improved false negative rate in Table 1 compared to Table 2 comes at the expense of a very large number of false positives. On the basis of the above simulations we recommend the use of BIC1.0. This version of BIC seems to have good control of the false positive error rate, a fact also noticed by [13]. The ratio m/n then appears to determine the false negative rate and our main source of error will be the inability to detect relationships as opposed to wrongly detecting non existent relationships.

### Estimating the non-zero elements of sigma inverse

Given the estimate of *B *in (6) above, we can write the log likelihood function as

(9) $$L=\left(n/2\right)\left\{\log\;\det\left(\Sigma {\left(\theta \right)}^{-1}\right)-trace\left(\Sigma {\left(\theta \right)}^{-1}S\right)\right\}$$

where *S = R^T ^R/n *and $R=X-D\hat{B}$. When the parameters *θ *correspond to the nonzero elements of Σ^-1^, differentiating L with respect to theta and equating to zero we see that the likelihood equations can be expressed as

(10) $$\sigma_{ij}=s_{ij}\;\text{if}\;\sigma^{ij}\neq 0$$

where, for example, *σ_ij _*denotes the ij^th ^element of Σ and *σ^ij ^*denotes the ij^th ^element of Σ^-1^, for details see [2] and (Kiiveri H and deHoog F: Fitting very large sparse Gaussian graphical models, submitted). From (10) we see that, at the maximum likelihood estimate, the elements of the estimated covariance matrix must be equal to those of the sample covariance matrix *S *whenever there is an edge between i and j in the graph and the elements of the estimated *inverse *covariance matrix must simultaneously be zero whenever there is no edge between i and j in the graph.

A necessary and sufficient condition for existence of the maximum likelihood estimate is that the sample covariance matrices restricted to the variables in the cliques of the graph associated with the model are all positive definite, [3]. This is almost certainly true provided the clique sizes are small relative to the sample size.

Solving the likelihood equations requires special care when p is large. Sparse matrix representations are required to minimise memory requirements. We use the methods of (Kiiveri H and deHoog F: Fitting very large sparse Gaussian graphical models, submitted) to obtain maximum likelihood estimates for the high dimensional setting in this paper.

### Significance testing

Testing hypotheses about *B *should really take into account the correlations between gene expression measurements. In this section we consider how to do this. Our tests will be conditional on the fitted (inverse) covariance matrix.

Beginning with (1), suppose we have an r × s matrix of orthogonal contrasts *C*, with 1 ≤ s < r and we wish to test the hypotheses

(11) $$C^{T}B\;=\;0$$

For example, in a study with n treatment subjects and m control subjects, we might have

$$D=\left[\begin{array}{cc} 1_{n} & 0\\ 0 & 1_{m} \end{array}\right]$$

where 1_k _is an k × 1 vector of ones. A contrast matrix of interest in this case with s = 1 might be $C^{T}=\left[1,-1\right]/\sqrt{2}$ in which case we are interested in testing for treatment differences relative to controls.

We can re-parameterise the problem so that (11) corresponds to testing for zero values in a new parameter matrix as follows. Expand C into an orthogonal matrix Q such that

$$Q\;=\;\left[AC\right]\;=\;\left[Q_{1}Q_{2}\right]$$

where *A *is r × (r-s). From (1) we can write

(12) $$\begin{array}{l} DB\;=\;DQQ^{T}B\\ \;\;\;\;\;\;=\;\tilde{D}\Gamma \end{array}$$

where $\tilde{D}=DQ$ and Γ = *Q^T^B*. If we partition Γ and $\tilde{D}$ conformably with Q so that $\tilde{D}=\left[{\tilde{D}}_{1}{\tilde{D}}_{2}\right]$ and Γ = [Γ_1 _Γ_2_], then (11) now corresponds to Γ_2 _= 0 in our new parameterisation.

From the result that

(13) $$\begin{array}{l} \hat{\Gamma }={\left({\tilde{D}}^{T}\tilde{D}\right)}^{-1}{\overset{⌢}{D}}^{T}X\\ vec\{\hat{\Gamma }\}=({\left({\tilde{D}}^{T}\tilde{D}\right)}^{-1}{\tilde{D}}^{T}\;⊗I)vec\{X\} \end{array}$$

it follows that $vec(\hat{\Gamma })$ has distribution

(14) $$N(vec\{\Gamma \},{\left({\tilde{D}}^{T}\overset{⌢}{D}\right)}^{-1}⊗\Sigma )$$

Under the Null hypothesis, ${\hat{\Gamma }}_{2}$ has distribution

(15) $$N(vec\{0\},G^{22}⊗\Sigma )$$

where 0 is an s × p matrix of zeroes and $G^{-1}={\left({\tilde{D}}^{T}\tilde{D}\right)}^{-1}$ is partitioned as

(16) $$\left[\begin{array}{cc} G^{11} & G^{12}\\ G^{21} & G^{22} \end{array}\right].$$

From (15) we define our test statistic for the null hypothesis Γ_2 _= *C^T^B *= 0 to be

(17) $$\begin{array}{c} T=vec{\left({\hat{\Gamma }}_{2}\right)}^{T}{\left(G^{22}⊗\Sigma \right)}^{-1}vec({\hat{\Gamma }}_{2})\\ =\;vec{\left({\hat{\Gamma }}_{2}\right)}^{T}vec({\left(G^{22}\right)}^{-1}{\hat{\Gamma }}_{2}\Sigma^{-1})\\ =\;trace\{{\left(G^{22}\right)}^{-1}{\hat{\Gamma }}_{2}\Sigma^{-1}{\hat{\Gamma }}_{2}^{T}\} \end{array}$$

Now writing (*G*^22^)^-1 = ^*LL^T^*, we can write (17) as

(18) $$T=trace({\tilde{\Gamma }}_{2}\Sigma^{-1}{\tilde{\Gamma }}_{2}^{T})$$

Where ${\tilde{\Gamma }}_{2}=L^{T}{\hat{\Gamma }}_{2}$. The diagonals in the trace can be decomposed as follows. Let γ = (*γ*_1_, *γ*_2_,....,*γ_p_*)*^T ^*denote the k^th ^row of ${\tilde{\Gamma }}_{2}$ then

(19) $$\begin{array}{c} \gamma^{\tau }\Sigma^{-1}\gamma =\sum_{i=1}^{p}\gamma_{i}{\left(\Sigma^{-1}\gamma \right)}_{i}\\ =\sum_{i=1}^{p}\gamma_{i}\sigma^{ii}(\gamma_{i}-\sum_{j^{\in n(i)}}\beta_{ij}\gamma_{j})\\ =\sum_{i=1}^{p}\sigma^{ii}\gamma_{i}{\left(\gamma_{N}\right)}_{i} \end{array}$$

where *β_ij _= -σ^ij^/σ^ii^, n(i)*denotes the neighbours of variable (gene) i and ${\left(\gamma_{N}\right)}_{i}=\gamma_{i}-\sum_{j^{\in n(i)}}\beta_{ij}\gamma_{j}$ are the neighbour corrected contrast values.

We could attempt to use the chi squared distribution to derive significance levels for *T*, however, due to the fact that Σ will be estimated and most likely contains specification errors, we will instead use permutation distributions.

Motivated by [5] we propose the following procedure for generating permutation distributions..

1. Fit the mean model under the Null hypothesis that Γ_2 _= 0. This gives fitted values

(20) $$\hat{F}={\tilde{D}}_{1}{\left({\tilde{D}}_{1}^{T}{\tilde{D}}_{1}\right)}^{-1}{\tilde{D}}_{1}^{T}X={\tilde{D}}_{1}\hat{\Gamma }$$

2. Compute the residuals R under the null hypothesis

$$R=X-\hat{F}.$$

3. For k = 1,..,q, generate new data sets *X*(*k*) according to

$$X(k)=\hat{F}+P_{k}R$$

where each *P_k _*is a randomly chosen permutation matrix

4. Compute $\hat{\Gamma }(k)$ using (13), namely

(21) $$\hat{\Gamma }(k)={\left({\tilde{D}}^{T}\tilde{D}\right)}^{-1}{\hat{D}}^{T}X(k)$$

5. Build up an empirical null distribution of T in (17) above using the ${\hat{\Gamma }}_{2}(k)$ from step 4 above. At the same time we can also build up empirical null distributions for each of the components of Γ_2 _and Γ_2*nb *_= Σ^-1^Γ*^T ^*which make up the test statistic T in (17).

It is easy to see that for our model the residuals in step 2 above have distribution

(22) $$vec\{R\}\;∼\;N(vec\{0\},(I-{\tilde{D}}_{1}{\left({\tilde{D}}_{1}^{T}{\tilde{D}}_{1}\right)}^{-1}{\tilde{D}}_{1})⊗\Sigma )$$

Hence for any permutation matrix *P_k _*we have

(23) $$vec\{P_{k}R\}\;∼\;N(vec\{0\},(I-P_{k}{\tilde{D}}_{1}{\left({\tilde{D}}_{1}^{T}{\tilde{D}}_{1}\right)}^{-1}{\tilde{D}}_{1}P_{k}^{T})⊗\Sigma )$$

When ${\tilde{D}}_{1}=c1_{n}$ it can be seen that (22) and (23) are identical. Such is the case, for example, when testing for the equality of the means of all groups in a study, see below. More generally, if the elements of *D *are bounded and (*D^T^D*)/*n*→*V *for some positive definite matrix *V *as n tends to infinity then (23) tends to (22) as n tends to infinity since

(24) $$\begin{array}{c} {\tilde{D}}_{1}{\left({\tilde{D}}^{T}{}_{1}{\tilde{D}}_{1}\right)}^{-1}{\tilde{D}}_{1}^{T}=D_{1}{\left(D^{T}{}_{1}D_{1}\right)}^{-1}D_{1}^{T}\\ =n^{-1/2}D_{1}{\left(D^{T}{}_{1}D_{1}/n\right)}^{-1}D_{1}^{T}n^{-1/2} \end{array}$$

To avoid complicated notation, in (24) above we have omitted a subscript n on *D*_1 _taking the dependence on n as understood.

In this paper the use of the normal distribution can be regarded as convenient way of keeping track of linear operations on means and covariances. However the results can all be interpreted as simply depending on means and covariances i.e. first and second order moments independent of specific distributional assumptions.

Testing the components of Γ_2*nb *_= Σ^-1^Γ*^T ^*is a new element which for want of a better term might be called testing for *differential connection*. Testing the individual components of Γ_2 _is analogous to testing for differential expression.

Null distributions for the individual components of the first equation in (19) can also be derived by permutation to test for significantly large sub components. The method described above can also be used to generate null distributions for the two components involving *γ *in the last equation of (19). Testing for the second component ((*γ_N_*)*_i _*is the new element due to the correlation between genes. To understand this second component, from equations (14) to (18) above, in the non null case, we can write

$$\begin{array}{l} \;\;\;\;\;\gamma \;∼\;N(\gamma_{0},\Sigma )\\ \Sigma^{-1}\gamma \;∼\;N(\Sigma^{-1}\gamma_{0},\Sigma^{-1}) \end{array}$$

where $\gamma_{0}^{T}$ is the k^th ^row of *L^T^*Γ. Some simple calculations show that

(25) $${\left(\gamma_{N}\right)}_{i}\;∼\;N(\gamma_{0i}-\;\sum_{j\in n(i)}\beta_{ij}\gamma_{0j},1/\sigma^{ii})$$

where, for example, *γ*_0*i *_is the i^th ^component of *γ*_0_. Under the null hypotheses *γ*_0 _= 0, or equivalently, Σ^-1^*γ*_0 _= 0 the mean in (25) is zero. If this hypothesis is rejected then the expected contrast value at gene i is not "smooth" i.e. a specified weighted linear combination of neighbouring contrast values. We have termed such a case differential connection. Intuitively, if nothing "unusual" is happening local to a specific gene, then we expect its contrast value to be roughly equal to a weighted linear combination of its neighbouring contrast values.

Note that testing for no differential expression can be regarded as testing a hypothesis concerning the marginal distribution of a particular gene whilst testing for no differential connection is testing a hypothesis concerning the conditional distribution of a gene given all the other genes, see the section on mixed graphical models in the supplementary information [Additional file 1] information for more details.

A simple example of using differential expression and differential connection to generate hypotheses for further investigation is given in the supplementary information [Additional file 1].

### A two group example

We illustrate some of the ideas above with an example involving n1 samples from a treatment group and n2 samples from a control group where the interest is in testing for equality of the treatment and control group means. Here the design matrix D, after suitable reordering can be represented as

$$D\;=\;\left[\begin{array}{cc} 1_{n1} & 0\\ 0 & 1_{n2} \end{array}\right]$$

and the contrast matrix C is defined as $C^{T}=[1,-1]/\sqrt{2}$. The orthogonal completion *Q *is

$$Q=\frac{1}{\sqrt{2}}\left[\begin{array}{rr} 1 & 1\\ 1 & -1 \end{array}\right]$$

The matrix $\tilde{D}$ is

$$\tilde{D}=\frac{1}{\sqrt{2}}\left[\begin{array}{rr} 1_{n1} & 1_{n1}\\ 1_{n2} & -1_{n2} \end{array}\right]$$

Writing N = n1 + n2, we also have

$$\begin{array}{c} I-{\tilde{D}}_{1}{\left({\tilde{D}}^{T}{}_{1}{\tilde{D}}_{1}\right)}^{-1}{\tilde{D}}_{1}=I-1_{N}1_{N}^{T}/N\\ \hat{F}=1_{N}1_{N}^{T}X/N\\ R=(I-1_{N}1_{N}^{T}/N)X\\ P_{k}R=P_{k}X-1_{N}1_{N}^{T}X/N \end{array}$$

Hence it can be seen that step 3 of the method for generating permutation distributions given above simply involves permuting the rows of X. The statistic T in (17) is a scalar multiple of ${\left({\hat{\beta }}_{1}-{\hat{\beta }}_{2}\right)}^{T}\Sigma^{-1}({\hat{\beta }}_{1}-{\hat{\beta }}_{2})$ where for example ${\hat{\beta }}_{1}$ is the p × 1 vector of sample means for the treatment group.

### Example

We use the smoking data of [21], with n = 57 subjects and p = 22283 gene expression measurements. There are two classes 34 smokers and 23 non smokers. We used the zero pattern finding strategy (with BIC_1.0_) defined earlier to determine the zero pattern in the inverse covariance matrix. For the regressions, the maximum neighbour size for each gene was restricted to 3 giving a ratio of observations per parameter (m/n) of approximately 1/20. The actual neighbour size distribution had minimum value 0, maximum value 26 and 90% of the neighbour sizes were in the range 1 to 6. The clique size distribution is given in Table 3 below

**Table 3:** Clique size distribution

clique size	1	2	3
count	202	32400	76

Using 20000 permutations of the rows of the data matrix *X *we obtained the null distribution of the test statistic $T\propto \;d^{T}{\hat{\Sigma }}^{-1}\text{​}d$ for testing the equality of the two class means, where d is the vector of differences of the means of the two classes. The value of the test statistic for our data was 3077.7. The quantiles of the null distribution were Table 4

**Table 4:** Quantiles of null distribution for T statistic in (17) for smoking data example

percentile	90%	95%	99%	99.995%
quantile	976.7	1023.7	1140.3	1491.6

so we can see that test value is highly significant leading us to conclude that the mean expression values of the smokers and non smokers are different. A histogram of the null distribution is given in Figure 4 below.

**Figure 4:**
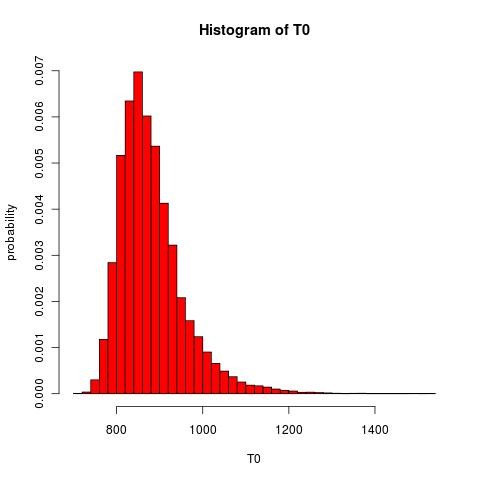
**Histogram of null distribution**.

We also derived null distributions for the two components of the last equation in (19). Testing the first of these is equivalent to testing for differential expression in this case.

We used the modified Bonferroni procedure of [22] to adjust for multiple testing. When testing m hypotheses the usual Bonferroni procedure with parameter *α *rejects all hypotheses whose p values is less than *α */*m *where 0 <*α *< 1. The modification of [22] allows, *α *> 1 in which case *α *is an upper bound on the expected number of false rejections i.e. false positives. This procedure exhibits strong control of the per family error rate under any dependence between p values. For details and a comparison with the Benjamini-Hochberg procedure see [22]. In setting the *α *parameter here, we note that we are performing approximately 40000 tests, and if we are willing to accept an expected number of false positives of 8 (say roughly 4 for each of the tests involving the *γ_i _*and the (*γ_N_*)*_i_*) the significance level to use in the tests is 8/40000 = .0002.

Applying the above procedure, 339 differentially expressed genes were identified and 1372 differentially connected genes were identified. Of the 97 genes identified by [21] as being differentially expressed using t-tests, 82 were also identified as differentially expressed using permutation tests and the procedure described above.

**Figure 5:**
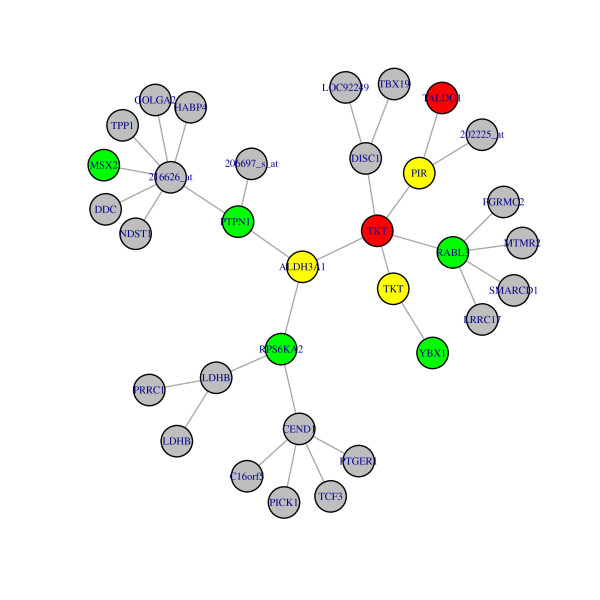
**Neighbourhood of size r = 3 around the gene ALDH3A1**. Grey denotes non- differentially expressed genes (probesets), red differentially expressed genes, green differentially connected genes and yellow genes which are both differentially expressed and differentially connected.

With this analysis, any gene of interest can be displayed in the context of a gene network. Define a neighbourhood of size r for any particular gene to be the set of genes which can be reached from this gene by traversing r edges in the associated graph derived from Σ^-1^. For example, a neighbourhood around each differentially expressed gene could be generated to identify interesting relationships. Figure 5 below shows the neighbourhood of size 3 of the differentially expressed gene ALDH3A1.

A related method for constructing local networks near genes of interest is given by [23]. Its focus is on creating local networks near a gene of interest, however, unlike the method described in this paper, it does not provide a joint model for the data or even a locally consistent model i.e. a positive definite covariance matrix. Note that Figure 5 is derived from a global consistent model.

**Figure 6:**
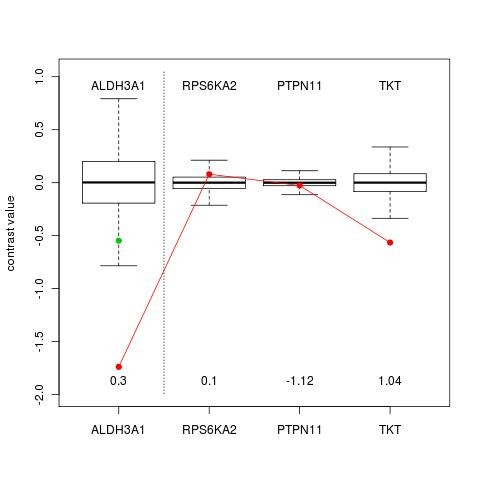
**Neighbours of differentially connected gene ALDH3A1**. The red dots denote the observed values of the contrast for the specified genes. The green dot is the predicted contrast value values based on the neighbours of ALHD3A1.

The gene ALDH3A1 is also differentially connected. Figure 6 below displays the relationship of this gene to its neighbours RPS6KA2, PTPN11 and TKT. In the plot the null distributions for the contrasts for each of the genes is presented as a boxplot.

The red line joins the actual observed values of the contrast between smokers and non smokers for each of the genes. The genes to the right of the dotted line are the predictor genes for ALDH3A1. The associated regression coefficients are given at the bottom of the plot. The value 0.3 is the variance of the error in the regression model. Note that the actual observed weighted average of the contrasts of the predictor genes is much lower than expected

From this plot the role of TKT and possibly RPS6KA2 in the expression of ALDH3A1 in smokers and non smokers needs to be investigated. Other explanations for this result such as post transcriptional processes may also be suggested.

Restricting attention to the differentially expressed genes, the subgraph defined by these genes has 247 clusters of connected genes. Table 5 below gives the distribution of these clusters by size of cluster.

**Table 5:** Distribution of clusters of differentially expressed genes

cluster size	1	2	3	4	5	6	8	9	17
no of clusters	210	22	5	3	2	1	2	1	1

**Figure 7:**
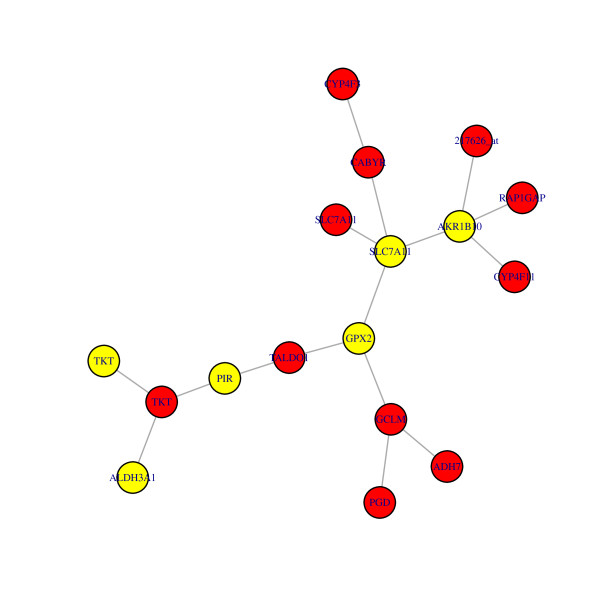
**Plot of genes in largest cluster of differentially expressed genes**. Red denotes genes which are differentially expressed and yellow denotes genes which are both differentially expressed and differentially connected.

Figure 7 below displays the genes and their connections in the largest cluster. Note that connections to non-differentially expressed genes are not shown.

A literature search revealed that all but 2 of the genes in Figure 6 are known to be connected. Some of the functions of genes in this sub network are xenobiotic metabolism, (ALHD3A1, ADH7, CYP4F11) and regulation of oxidant stress and glutathione metabolism(TALDO1, PGD, GPX2,CYP4F3), known to be important in cigarette smoking, see [21].

Graphical queries such as which is the closest differentially expressed gene to a specified gene and which is the shortest pathway between two given genes can also be answered.

Mixed graphical models based on trees and forests have also been used by [24] to analyse microarray data. Software for their approach is described in [25].

## Discussion

The parameter kmax in the strategy for determining the pattern of zeroes would not be necessary if we had large sample sizes. Unfortunately, in practise, this is not usually the case. Simulations in this paper and **(**Kiiveri H and deHoog F: Fitting very large sparse Gaussian graphical models, submitted) support the general conclusion that more connections are detected and the bias and variability in the estimates of Σ^-1 ^is reduced when the ratio kmax/n decreases. Based on limited evidence to date, a tentative upper limit on this ratio would be about 1/20 which corresponds to 20 observations per parameter in the largest regression models. It is easy to see that for any given example, a model derived from a smaller value of kmax will produce a sub-model of one derived from a larger kmax. Determining the pattern of zeroes and fitting Σ^-1 ^will typically be faster for smaller values of kmax. There are also other reasons one might wish to limit the size of kmax. For example, as a preliminary exploratory analysis, it would not be unreasonable to look for only a few of the strongest connections between genes to ovoid being overwhelmed by large numbers of network connections.

The inclusion of correlations into the analysis via sparse inverse covariance matrices comes at a substantially increased computational cost. The three main computational steps are

(i) identifying the pattern of zeroes in the inverse covariance matrix

(ii) fitting the inverse covariance matrix and

(iii) computing permutation distributions

Steps (i) and (iii) can be done in a day or so on a single desktop machine. A single lars stepwise fit stopped at kmax terms requires O(npkmax + kmax^3^) operations and the computation of (22) requires O(nr^2 ^+ p(nr + r^2^)) operations, see [19] and [[26], p240]. However these steps are also very easy to parallelise and so can be speeded up with very little effort if more processors are available. Depending on the structure of sigma inverse, step (ii) can also be performed in a day or less. However, there are cases when this step is more difficult and can take longer. We are currently exploring ways of parallelising this calculation as well as a promising alternative optimisation method. Another approach, given a pattern of non-zeroes in sigma inverse, could be to estimate the elements of Σ^-1 ^simply by regression i.e. a regression of each gene on its neighbours. The equations $\beta_{ij}=-(\sigma^{ij}/\sigma^{ii}),\;v_{i}^{2}=1/\sigma^{ii}$ where *β_ij _*is the regression coefficient of gene i on gene j, and $v_{i}^{2}$ is the residual variance for the regression then provide a means for "estimating" the elements of Σ^-1^. However such an analysis would at best be an approximation since the estimated Σ^-1 ^may not be exactly symmetric, nor positive definite. None the less it could be a method worth exploring as such a method would be asymptotically consistent if the neighbour structure was correct.

The advantage of the maximum likelihood estimate of sigma inverse is the possibility of doing simulations, for example of the likely effects of controlling the expression of specified genes.

The method can generate many interesting hypotheses involving the connections between genes, explanations for differential expression in terms of neighbouring genes and connected pathways, and places in the network where connected genes are acting unusually.

Note that the extension to the case that the contrast matrix C in (11) is full rank rather than orthogonal is straightforward.

R code implementing the methods of this paper is freely available in the library mvama, see [27].

## Conclusion

There is a wealth of information about relationships between genes in a microarray experiment which is currently underutilised. In this paper we have present a practical strategy for accessing some of this information. We have presented a method for incorporating correlations between genes into the analysis of microarray data. A by-product is a method for the analysis of differential expression which does not require the empirical Bayes model of the traditional approach of [1] nor any need to estimate the number of differentially expressed genes a priori. The new approach produces a gene network for all the genes and allows differential expression to be placed within the context of the gene network.

### Future work

In future work we hope to consider transformations of expression data to make it have an approximate multivariate normal distribution, a comparison of different methods for identifying the pattern of zeroes in sigma inverse and faster algorithms for fitting the inverse covariance matrix.

## Methods

### Relationships between matrices stacked row by row and column by column

Suppose *A, B*, *X and Z *are matrices such that the appropriate matrix products below are well defined. Let *vec{X} *denote the vector obtained from the matrix *X *by stacking *rows *sequentially beginning with row 1. Let *vecc{X} *denote the vector obtained from *X *by stacking *columns *in a similar way. It is easy to see that

(26) $$vec\left\{X\right\}=vecc\left\{X^{T}\right\}$$

(27) $$vecc\left\{X\right\}=vec\left\{X^{T}\right\}$$

An important identity [8] which we use in this paper is

(28) $$\left(A⊗B)vec\{X\}=vec\{AXB^{T}\right\}$$

Note the special cases when *A = I *or *B = I*. Using (26), from (28) we can see that

$$\left(A⊗\;B)vecc\{X^{T}\}=vecc\{BX^{T}A^{T}\right\}$$

Replacing *X*^T ^by *Z *gives us the equivalent identity

$$(\text{A}\;⊗B)vecc\{Z\}=vecc\{BZ{\text{A}}^{T}\},$$

see equation (5) in [7].

Another useful identity is

$$\begin{array}{c} vec{\left\{A\right\}}^{T}vec\{B\}=vecc{\left\{A^{T}\right\}}^{T}vecc\{B^{T}\}\\ =trace\{AB^{T}\}=trace\{BA^{T}\} \end{array}$$

## Supplementary Material

Additional file 1 **Supplementary information**. Click here for file
