# Supplementary material for: Multivariate analysis of microarray data: differential expression and differential connection
Source: BMC Bioinformatics. 2011 Feb 1;12:42. doi: 10.1186/1471-2105-12-42 (PMC3045302; doi:10.1186/1471-2105-12-42)
Supplement: Additional file 1 — Supplementary information. [file 1471-2105-12-42-S1.PDF]

Multivariate analysis of microarray data:

differential expression and differential connection

Supplementary information

Harri T Kiiveri

CSIRO Mathematics Informatics and Statistics, The Leeuwin Centre, 65 Brockway Road, Floreat, Western Australia

Email addresses:

HTK: harri.kiiveri@csiro.au

Abstract

Background

Typical analysis of microarray data ignores the correlation between gene expression values. In this paper we present a model for microarray data which specifically allows for correlation between genes. As a result we combine gene network ideas with linear models and differential expression.

Results

We use sparse inverse covariance matrices and their associated graphical representation to capture the notion of gene networks. An important issue in using these models is the identification of the pattern of zeroes in the inverse covariance matrix. The limitations of existing methods for doing this are discussed and we provide a workable solution for determining the zero pattern. We then consider a method for estimating the parameters in the inverse covariance matrix which is suitable for very high dimensional matrices. We also show how to construct multivariate tests of hypotheses. These overall multivariate tests can be broken down into two components, the first one being similar to tests for differential expression and the second involving the connections between genes.

Conclusion

The methods in this paper enable the extraction of a wealth of information concerning the relationships between genes which can be conveniently represented in graphical form. Differentially expressed genes can be placed in the context of the gene network and places in the gene network where unusual or interesting patterns have emerged can be identified, leading to the formulation of hypotheses for future experimentation.

A forward stepwise regression algorithm

Let the n x p matrix X and the n x 1 response vector y be mean corrected. We write X_s for the submatrix of X defined by columns in the set S, and $\hat{\beta}_s$ for the regression coefficients computed from the regression of y on X_s. The algorithm can then be described as follows.

1. Begin with the regression model having no predictors, S $=\emptyset$ and let r=y.
2. Find the column of X which has maximum correlation with r.
3. Add this column to the regression model, update S and compute the residuals $r = y - X_s\hat{\beta}_s$
4. Repeat steps 2 to 3 until the number of predictors equals n-1 or the rank of X.

Mixed graphical models

Mixed graphical models are a convenient way to represent many common statistical models (as well as generating new ones) and to understand the implications of various constraints placed on the parameters in these models. In this section we briefly present some details on mixed graphical models (Edwards (1990,2000)) and their connection with the model in the main paper. To minimise notation clashes with the current paper we make some changes in the standard notation for mixed graphical models.

Mixed graphical models are defined through a parameterisation of the so called conditional Gaussian (CG) distribution as follows. Consider q discrete variables taking values $d = (d_1,...,d_q)^T$ with $d_k \in \{1,2,...,r_k\}$ for k=1,…,q, and p continuous Gaussian variables taking values $x = (x_1,...,x_q)^T$. The joint density of these variables can be written as

$$f(d,x) = p(d)(2\pi)^{-p/2}\exp\{-\frac{1}{2}(x-\mu(d))^T\Sigma^{-1}(d)(x-\mu(d))\} \qquad \text{(A.1)}$$

where *p(d)* is the marginal density of the discrete variables. Note that (A.1) specifies a multivariate normal distribution with mean $\mu(d)$ and covariance matrix $\Sigma(d)$ for each cell of a contingency table defined by the discrete variables. Equation (A.1) can be written in exponential family form as

$$f(d,x) = \exp\{\alpha(d) + \beta(d)^T x - \frac{1}{2}x^T\Omega^{-1}(d)x\} \qquad \text{(A.2)}$$

Equation A.1 is termed the moment parameterisation of the CG distribution and (A.2) is termed the canonical or “natural” parameterisation. Each of the canonical parameters can be given an expansion in terms of interaction terms. The relationships between the parameterisations are given by

$$\begin{aligned}\beta(d) &= \Sigma^{-1}(d)\mu(d)\\ \Omega(d) &= \Sigma^{-1}(d)\end{aligned}. \qquad \text{(A.3)}$$

For the connection between *p(d)* and *α(d)*, interaction parameterisations of the canonical parameters and the graphical representation of these models, see Edwards(1990,2000).

From (A.1) we can easily calculate the conditional distribution of x_i given $x_{-i} = \{x_j : j \neq i\}$ and d as

$$f(x_i \mid x_{-i}, d) = N(\beta_i(d)/\sigma^{ii}(d) + \sum_{j \neq i} b_{ij}(d) x_j \;, 1/\sigma^{ii}(d)) \qquad \text{(A.4)}$$

Where N denotes the univariate normal distribution, β is defined in (A.3) and $b_{ij}(d) = -\sigma^{ij}(d)/\sigma^{ii}(d)$. Hence we can see that setting $\beta_i(d)$ to zero in (A.2) corresponds to the first term in the mean of (A.4) being zero. If in addition Σ^{-1} does not depend on d there is no dependence on d in (A.4).

For the smoking data example in the main paper we have one discrete variable, namely the smoking status variable so q=1 in the above. We have p=22283 X variables corresponding to the expression values for each gene. The covariance matrix Σ is restricted to be independent of d i.e the same for smokers and non smokers. The model density is derived by conditioning on the smoking variable i.e we calculate $f(x \mid d_1)$ from (A.1). More generally, when the design matrix is defined by discrete factors and continuous variables x_1 (say), partitioning x as $(x_1^T, x_2^T)^T$ we would use $f(x_2 \mid x_1, d)$.

For the smoking data, the mean model can be represented as

$$\mu(1) = \lambda + \gamma(1)$$
$$\mu(2) = \lambda + \gamma(2)$$

where, for example, $\mu(1)$ is the population mean for non smokers and $\gamma(1) + \gamma(2) = 0$. This last constraint implies that we can write $\gamma(1) = \gamma,\ \gamma(2) = -\gamma$ for some vector of parameters γ, representing the contrast between the mean of the smokers and non smokers. Using (A.3), the corresponding structure on the beta parameters in the canonical parameterisation (A.2) is of the same form

$$\beta(1) = \lambda^* + \gamma^*(1)$$
$$\beta(2) = \lambda^* + \gamma^*(2) \qquad \text{(A.5)}$$

where $\lambda^* = \Sigma^{-1}\lambda$ and $\gamma^*(k) = \Sigma^{-1}\gamma(k)$, for k=1,2. This property is known as mean linearity. For the smoking data model, Σ^{-1} is a sparse matrix with many zeroes and has no dependence on d. Hence substituting (A.5) into (A.4) we obtain for d=1,2

$$f(x_i \mid x_{-i}, d) = N(\{\lambda_i^* + \gamma_i^*(d)\}/\sigma^{ii} + \sum_{j \neq n(i)} b_{ij} x_j \;, 1/\sigma^{ii}) \qquad \text{(A.6)}$$

where n(i) denotes the set of neighbours of gene i.

Hence a hypothesis about an element of the neighbour adjusted contrast is a hypothesis in the conditional distribution (A.4) of which (A.6) is a special case. This can be compared with equation (25) in the main paper. From (A.6) we can see that the hypothesis of no differential connection for gene i implies the conditional independence of gene i from the smoking variable, given its neighbours.

The hypothesis of no differential connection can be conveniently represented graphically. Suppose we have three genes A, B and C from our smoking study and we represent the binary smoking variable by D. In addition suppose A and C are differentially connected but B is not. Then the subgraph of the model restricted to these variables might be as in Figure S1 below.

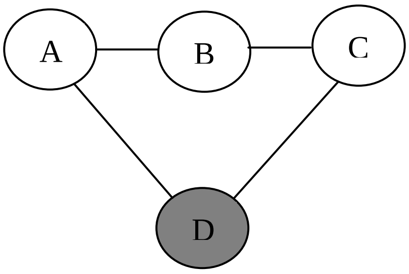

Figure S1: Example of graphical representation of no differential connection

No differential connection corresponds to the absence of an edge between D and B. The absence of an edge between A and C corresponds to a zero on the common inverse covariance matrix of A,B and C. For more details about the graphical representation of these models see Edwards(1990,2000).

Example of hypothesis generation

Suppose the (local) graph of the model contains elements as depicted in Figure S2 below. For simplicity we omit connections to other genes. Here genes A and C are differentially expressed and Gene B differentially connected but not differentially expressed.

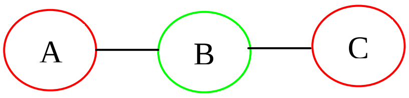

Figure S2: Example graph of hypothetical sub network

If we consider all possible causal connections between genes A, B and C in Figure S1 we obtain the four alternative graphs in Figure S3 below. Considering Figure S3, we might expect alternatives 1,3 and 4 to imply differential expression of gene B so we exclude them. In Alternative 2 we might hypothesize that gene B is involved in regulating Genes A and C e.g. it might be a transcription factor. Clearly there are other possibilities such as the effects of A and C cancelling each other out in Alternative 3. Checking the signs of the regression coefficients and the direction of differential expression could be useful here. Properly designed experiments would need to be carried out to determine what is really going on.

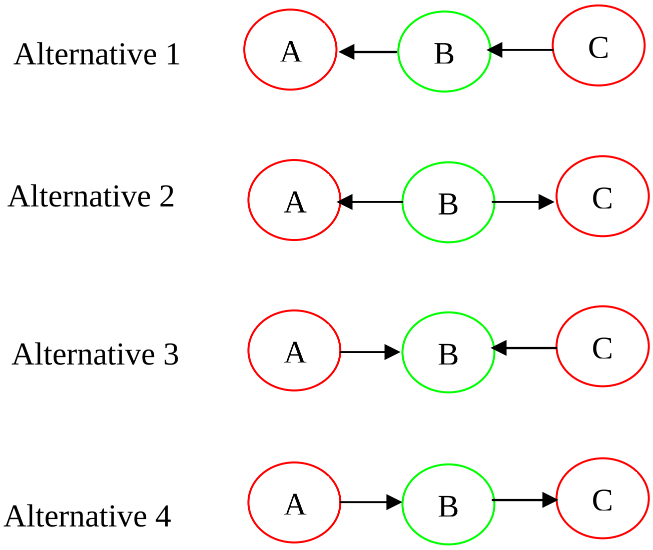

Figure S3: All possible causal connections between genes in Figure S2

References

Edwards, D: **Hierarchical Interaction models**. *J R Stat Socs B 1990,* **52** ,3-20.

Edwards, D: *Introduction to Graphical Modelling, second edition*, Springer-Verlag, New York 2000.
